# Microstructure-Based Thermochemical Ablation Model of Carbon/Carbon-Fiber Composites

**DOI:** 10.3390/ma15165695

**Published:** 2022-08-18

**Authors:** Xiaobin Wang, Peng Jiang, Yujian Tang, Weixu Zhang, Shengbo Shi

**Affiliations:** 1 State Key Laboratory for Strength and Vibration of Mechanical Structures, School of Aerospace Engineering, Xi’an Jiaotong University, Xi’an 710049, China; 2 China Nuclear Power Engineering Co., Ltd., Beijing 100142, China; 3 School of Astronautics, Northwestern Polytechnical University, Xi’an 710072, China

**Keywords:** carbon/carbon composites, thermochemical ablation model, microstructure, ablation recession rate

## Abstract

The microstructure of carbon fiber–reinforced carbon-matrix composites (carbon/carbon composites) has important effects on its ablation performance. However, the traditional macro-ablation methods have underestimated the ablation recession rate and ignored the influence of microstructure. To simulate the ablation of large-sized structures while accounting for the influence of microstructure, it is necessary to modify these methods. In this work, a thermochemical ablation model for carbon/carbon composites is proposed based on the evolution behavior of their microstructure. The ablation recession rate and surface temperature predicted by this model are in good agreement with the experimental results. Through numerical analysis, we found that the ablation recession rate of the material without carbon fibers is much greater than that of the material containing carbon fibers. The ablation recession rate is influenced by the fiber orientation due to the change in thermal conductivity. The anti-ablation efficiency of carbon/carbon composites can be improved by increasing their fiber radius, radiation coefficient, specific heat capacity, interphase density, and thermal conductivity coefficient. The thermochemical ablation model provides a guide for the design of better anti-ablation carbon/carbon composites.

## 1. Introduction

Carbon fiber–reinforced carbon-matrix composites (carbon/carbon composites), which possess high latent heat of sublimation, excellent strength, and chemical stability at high temperatures, have been widely used as thermal protection materials in atmospheric re-entry vehicle heat shields and rocket engine nozzles [1,2,3,4,5,6,7,8,9,10,11]. High-temperature, high-pressure, and high-velocity airflow are present in the typical service environments of carbon/carbon composites. Ablation, which is a material consumption behavior of carbon/carbon composite surfaces in service environments, is an effective protection mechanism for aircraft components [12,13,14]. The thermochemical ablation behavior of carbon/carbon composites involves the heat transfer, chemical reactions, surface recession and mass loss, which seriously affects some performance parameters of aircraft. Among them, the ablation recession rate is a key parameter for the characterization of surface recession behavior and the thickness design of thermal protection materials. With the progress of weaving technologies, the microstructure of carbon/carbon composites is becoming more and more complex. It changes not only the volume fraction of carbon fiber but also the fiber orientation and void volume fraction. Due to the different characteristics of the fibers and matrix [15,16], these microstructural changes have a great impact on the thermochemical ablation behavior. To reveal the ablation mechanisms, it is important to investigate the thermochemical ablation behavior of carbon/carbon composites at the micro scale.

Numerous researchers have focused on the thermochemical ablation behavior of carbon/carbon composites under high-temperature airflow [17,18,19], especially on its analysis models. These studies can generally be divided into the study of: (1) heat transfer analysis [20,21,22], (2) detailed chemical reactions [23,24,25], (3) surface ablation morphology [26,27], and (4) the effects of the material microstructure on ablation characteristics [28,29,30]. In the aspect of heat transfer analysis, the thermal response was simulated from the perspective of the matrix and fiber components of various braided carbon/carbon composites [31,32]. The results showed that the temperature distributions of the matrix and fiber, which presented different characteristics during ablation, were not uniform along the thickness direction of the material structure. In the aspect of detailed chemical reactions, the thermochemical ablation products were commonly studied based on the principle of minimizing the free energy and microscopic analysis by X-ray photoelectron spectroscopy (XPS) and X-ray diffraction (XRD) [33,34]. Thermogravimetric analysis (TGA) was used to determine the oxidation kinetics of carbon/carbon composites in oxidizing conditions [35,36]. Based on the analysis of detailed chemical reactions, the combined numerical analysis models of the fluid, thermal, and physicochemical reactions during the ablation process were established and used to predict/analyze the thermal response and ablation properties of the material [37]. Among them, the traditional macro-ablation model of physicochemical reactions assumes that the material surface is a flat plate and only considers the diffusion and reaction of gas in the boundary layer. It underestimates the ablation recession rate and ignores the influence of microstructure, which is inconsistent with the actual rough material surface. In the aspect of surface ablation morphology, the ablation microstructure of carbon/carbon composites has been studied by scanning electron microscopy (SEM) and microtomography [38,39]. These studies revealed the existence of a thin reactive interphase between the fibers and the matrix. The ablation recession rate of the interphase was higher than that of the fibers and the matrix. Based on the above phenomenon, Lachaud et al. [40] established a steady-state ablation morphology model for carbon/carbon composites to study the effects of reactivity and gas diffusion on the ablation morphology. The results might be beneficial for predicting of the surface roughness, predicting of the effective reactivity of the composite components and the inverting of the extremely harsh environment of composites. However, the morphology made it difficult to obtain specific data and to use as an index to evaluate the ablation performance of the materials, which limited the development of the morphology research. In the aspect of material microstructure, the effect of density and fiber orientation on the ablation behavior of carbon/carbon composites was investigated. The results showed that the fiber architecture and the density had more effects on the ablation behavior than the open porosity of the composites [41]. Liu et al. [42] investigated the impact of temperature and fiber volume fraction on the ablation performance of the carbon/carbon composites using numerical simulations. However, only the influence of the temperature was considered by the element exclusion criteria in this method, and the effect of gas diffusion and other factors were neglected. In fact, the temperature differential is very small at the microscopic scale. Li et al. [43] analyzed the influence of the braided angle, porosity, and reactivity contrast between the matrix and yarns on the mesoscopic thermochemical ablative behaviors. These works only considered the mesoscopic structure in the geometric modeling of materials but not in the theoretical model. It meant the models could only be used for material ablation simulation but not for large structures.

In summary, the thermochemical ablation recession rates of carbon/carbon composites are closely related to the properties and arrangement orientation of the components, the morphological characteristics of the reinforcing phase, and the interfacial properties between the reinforcing phase and the matrix. The traditional macro-ablation model only considers mesoscopic structure in geometric modeling, so it is impossible to consider the influence of detailed microstructure on ablation performance in large-sized structures. The microstructure such as fiber orientation has a significant effect on the ablation performance, it is necessary to modify the thermochemical ablation model to take the microscopic characteristics into account. Therefore, an effective microstructure-based thermochemical ablation analysis model for carbon/carbon composites is proposed in this work to study thermochemical ablation behavior.

This paper is structured as follows. In Section 2, we analyze the ablation mechanism and proposed a microstructure-based thermochemical ablation model of carbon/carbon composites. In Section 3, we introduce the calculation process of the model. The model was validated by comparison to the experimental data of Reference [25]. Section 4 discusses the effects of the microstructure, material properties and external environment on the surface ablation recession rate. Section 5 concludes the main results. The research law of microstructure may be applied to the prediction of the macro-ablation performance of ablative materials in this paper, and this work may provide a reference for the design and preparation of better high-ablation resistance carbon/carbon composites.

## 2. Thermochemical Ablation Model of Carbon/Carbon Composites

### 2.1. Problem Statement

**Figure 1:**
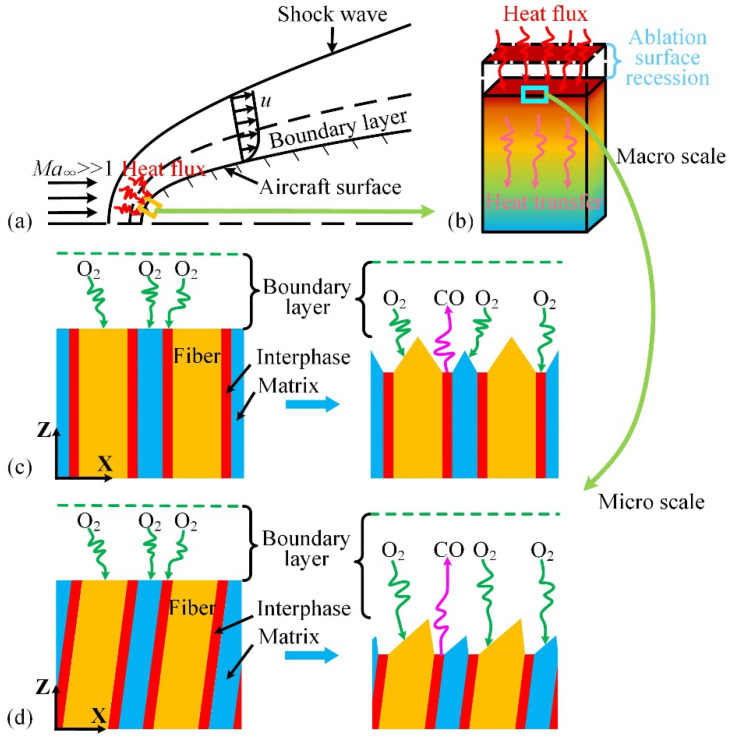
Schematic diagrams of (**a**) hypersonic flow around a bluff body; (**b**) heat transfer and ablation surface recession at the macro scale; (**c**) changes in the surface morphology of vertical fibers before and after ablation at the micro scale; and (**d**) changes in the surface morphology of inclined fibers before and after ablation at the micro scale.

While flying at ultra-high speed in the atmosphere, an aircraft interacts violently with the surrounding air, creating a strong shock wave ahead, as shown in Figure 1a. The airflow is sharply compressed after the shock wave. The kinetic energy of the airflow is converted into internal energy of the air molecules, which heats the airflow to an ultra-high temperature. The flow of heat in the air acts on the material surface through convective and radiative heat transfer, and a part of the heat is transferred to the material. This creates a temperature gradient inside the material in which the surface temperature is higher and the internal temperature is lower, as shown in Figure 1b. In a high-temperature airflow environment, the surface material reacts with the air in a complex manner and becomes very rough. This process is called thermochemical ablation, which results in quality loss and surface recession.

The carbon/carbon composite is a gasification-type ablation material that exhibits complex thermochemical ablation characteristics under high-temperature airflow. The thermochemical ablation of the carbon/carbon composites involves complex physicochemical reactions and gas diffusion, such as oxidation reactions, carbon–nitrogen reactions, sublimation, and oxidation diffusion, as shown in Equations (1)–(5):

(1) $$2C(s)+O_{2}(g)\;⇌\;2\mathrm{CO}(g),$$

(2) $$C(s)+O_{2}(g)\;⇌\;{\mathrm{CO}}_{2}(g),$$

(3) $$4C(s)+N_{2}(g)\;⇌\;2C_{2}N(g),$$

(4) $$2C(s)+N_{2}(g)\;⇌\;2\mathrm{CN}(g),$$

(5) $$iC(s)\;⇌\;C_{i}(g),\;i=1,\;2,\;3$$

CO(g) is the major ablation product of the carbon/carbon composites at temperatures below 3300 K because of the insufficient oxygen on the surface of the material [33]. Equation (1) describes a spontaneous chemical reaction at high temperatures; for example, because Δ*G*°(CO) = −654.726 kJ·mol^−1^ at 2500 K, this reaction can be considered as irreversible because the equilibrium partial pressure ratio between the reactant and product is $P_{\mathrm{CO}}^{2}/P_{O_{2}}=4.796\times 10^{13}$ Pa. Consequently, the thermochemical ablation model in this study considers only the CO(g) formation reaction.

**Figure 2:**
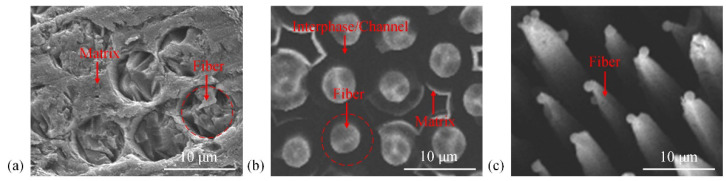
SEM images of carbon/carbon composite morphology: (**a**) before oxidation; (**b**) after oxidation at 1200 °C in air for 8 min; (**c**) after oxidation at 1200 °C in air for 15 min.

Figure 2 shows the typical morphology of carbon/carbon composites before and after oxidation in air. Based on the main characteristics of the fiber composites shown in Figure 2, we create an abstract analysis model shown in Figure 1c,d for the numerical simulation. The morphology of the channels in the SEM image indicates that ablation occurs at the fiber–matrix interface. In other words, the fiber–matrix interfaces are much easier to oxidize than the fiber and matrix. Under high-temperature airflow, the oxygen in the boundary layer impinges and reacts with the carbon on the material surface. Then, the CO gas product escapes from the material surface into the boundary layer. As the interphase continuously retreats, gas diffusion channels are formed. The channels expose the fibers and matrix to the heat airflow. As shown in Figure 1c, oxygen is transported from the boundary layer to the ablation surfaces through the gas diffusion channels. With the growth of the gas diffusion channels, the oxidation rate of the interphase is limited by gas diffusion. As a result, the thermochemical ablation mechanisms are diffusion-limited and/or reaction-limited. In addition, as shown in Figure 1d, the fiber orientation affects the fiber morphology and influences the gas diffusion.

### 2.2. Thermochemical Ablation Model

The thermochemical ablation mechanisms are diffusion-limited and/or reaction-limited. The ablation recession rate of the interphase *v*_i_ can be obtained from the reactivity *k*_i_ (m·s^−1^) and the molar concentration of oxygen *C*_i_ (mol·m^−3^) at the surface of the interphase [40]:

*v*_i_ = Ω_i_*J*_C_(*z* = 0), (6)

(7) $$J_{C}(z=0)=2J_{O_{2}}(z=0)=2k_{i}C_{i},$$

where Ω_i_ is the solid molar volume of interphase (m^3^·mol^−1^), *J*_C_ is the molar rate of ablation (mol·m^−2^·s^−1^). Similarly, the ablation recession rate of the fiber *v*_f_(*z*) can be written as

*v*_f_(*z*) = Ω_f_*J*_C_(*z*), (8)

(9) $$J_{C}(z)=2J_{O_{2}}(z)=2k_{f}C(z),$$

where the coordinates in the thermochemical ablation model are micro scale, while the coordinates in the one-dimensional thermal response model of Section 2.3 are macro scale.

In this work, the macro-ablation recession rate of the material *v*_macro_ is defined as the average ablation rate, and can be written as:

(10) $$v_{\mathrm{macro}}=\frac{\int_{0}^{t}v_{\mathrm{micro}}dt}{t}=\frac{\delta \left(t\right)-\delta_{0}}{t},$$

where *δ*(*t*) and *δ*_0_ are the sample thickness at time *t* and the initial thickness of specimen (m), respectively, and *t* is the ablation time (s). The micro-ablation recession rate *v*_micro_ can be written as:

(11) $$v_{\mathrm{micro}}=\{\begin{array}{c} v_{f}{(z=h}_{f}),\;\mathrm{when}\;\mathrm{the}\;\mathrm{top}\;\mathrm{of}\;\mathrm{the}\;\mathrm{fiber}\;\mathrm{morphology}\;\mathrm{presents}\;a\;\mathrm{platform}\\ v_{i},\;\mathrm{when}\;\mathrm{the}\;\mathrm{top}\;\mathrm{of}\;\mathrm{the}\;\mathrm{fiber}\;\mathrm{morphology}\;\mathrm{is}\;\text{needle-shaped} \end{array}.$$

The reaction to produce carbon monoxide can be viewed as a one-step reaction. Thus, the temperature-dependent reactivity *k* can be described by the first-order Arrhenius equation [44]:

*k* = *A*exp(−*Ea*/*RT*), (12)

where *A* is the pre-exponential factor (m·s^−1^), *Ea* is the activation energy of the reaction between carbon and oxygen generating carbon monoxide (J·mol^−1^), *R* is the universal gas constant (J·mol^−1^·K^−1^), and *T* is the temperature (K).

We assume that the oxygen transport can be described by pure diffusion. Thus, the mass conservation for the gas can be written as [45]:

∂*C*/∂*t* + ∇⋅(−*D*∇*C*) = 0, (13)

where *D* is the diffusion coefficient (m^2^·s^−1^). The mutual diffusion of oxygen and carbon monoxide is given by [46]:

(14) $$D=\frac{3}{8n{\left[\frac{1}{2}{(d}_{1\;}{+d}_{2})\right]}^{2}}{\left[\frac{{\mathrm{RT}(m}_{1}{+m}_{2})}{{2\pi m}_{1}m_{2}}\right]}^{\frac{1}{2}},$$

where *n* is the number density (m^−3^), *d*_1_ and *d*_2_ are the molecular diameters of O_2_ and CO (m), respectively, and *m*_1_ and *m*_2_ are the molar masses of O_2_ and CO (kg·mol^−1^), respectively.

It is assumed that the material quickly reaches a steady state at the microscopic scale, and that the oxygen concentration has a gradient only along the Z-direction. Hence, according to Equation (13), the oxygen concentration distribution *C*(*z*) can be written as:

(15) $$C(z)=C_{i}+\frac{C_{0}-C_{i}}{h_{f}+\delta_{c}}\;z,$$

where *δ*_c_ is the boundary layer thickness (m), *C*_0_ is the molar concentration of the boundary layer (mol·m^−3^), and *C*_i_ = *C*_0_/(1 + *Da*_i_), where *Da*_i_ = *k*_i_(*h*_f_ + *δ*_c_)/*D* is the Damköhler number [47].

The top of the fiber is flat at first. Therefore, the fiber height *h*_f_ can be written as:

(16) $$h_{f}=\int_{0}^{t}{[v}_{i}-v_{f}(z=h_{f})]dt.$$

**Figure 3:**
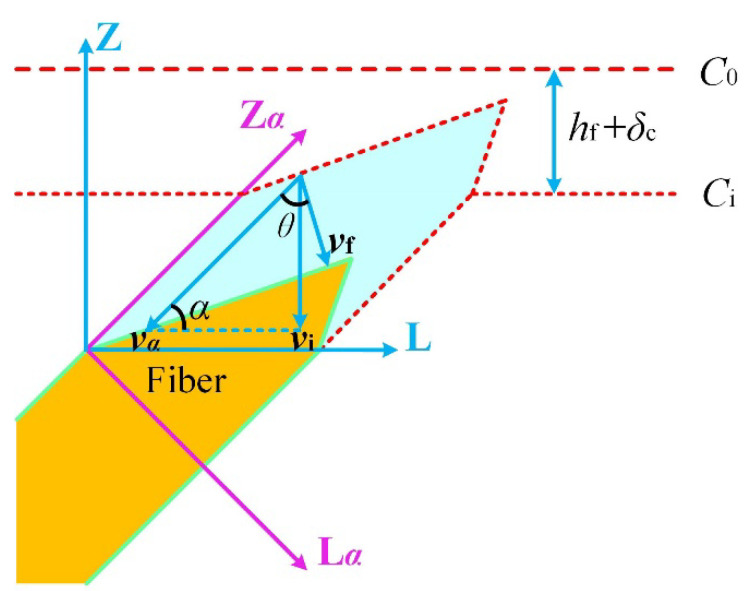
Schematic diagram of fiber morphology recession during steady-state ablation.

When the platform at the top of the fiber disappears, Equation (16) no longer works because the ablation recession velocity of the fiber is not parallel to the *Z*-axis. As shown in Figure 3, in the steady-state ablation process, the relationship between the ablation velocity of the inclined fiber and the interphase can be rewritten as:

*v*_f_ = *v_α_*cos* θ* = *v*_i_cos *θ*/sin *α*. (17)

The fiber surface morphology can then be described as:

(18) $$\frac{\partial l_{\alpha }}{\partial z_{\alpha }}=\pm \frac{1+\frac{z_{\alpha }\sin\alpha -l_{\alpha }\cos\alpha }{\chi }}{\sqrt{{\left(\frac{\eta }{\sin\alpha }\right)}^{2}-(1+\frac{z_{\alpha }\sin\alpha -l_{\alpha }\cos\alpha }{\chi }{)}^{2}}},$$

where *α* is the tilt angle (°), *z_α_* and *l_α_* are the coordinates of the fiber surface (m), *χ* = *D*/*k*_i_ is the characteristic length of diffusion (m) (which is related to the interphase reactivity and the gas diffusion), and *η* = (*k*_i_Ω_i_)/(*k*_f_Ω_f_) is a dimensionless number that represents the relative reactivity.

When the tilt angle *α* = 90°, *h*_f_ is given by the steady-state characteristic height:

(19) $$h_{f}=\{\begin{array}{c} \sqrt{{2l}_{f}\chi \sqrt{B^{2}-1}+\chi^{2}-l_{f}^{2}}-\chi ,\;l_{f}<\chi \sqrt{B^{2}-1}\\ \chi (B-1),\;l_{f}\geq \chi \sqrt{B^{2}-1} \end{array},$$

where *l*_f_ is the characteristic radius of the carbon fiber on the cross-section (m).

### 2.3. One-Dimensional Thermal Response Model

In the thermal response model, we have made appropriate simplifications. There are the following assumptions:

(a) The material surface is subjected to uniform heat flow, and the other surfaces satisfy the adiabatic boundary conditions.

(b) The temperature changes only in the sample thickness direction, that is, it satisfies the one-dimensional thermal response model.

(c) The residual resin pyrolysis in carbon/carbon composites can be neglected.

In the one-dimensional heat transfer problem, the energy conservation equation in the ablation material control body can be written as [48,49]:

(20) $$\rho C_{p}\frac{\partial T}{\partial t}=\frac{\partial }{\partial z}(\lambda \frac{\partial T}{\partial z}),$$

where, *ρ* is the density, *C*_p_ is the specific heat capacity, and *λ* is thermal conductivity coefficient.

**Figure 4:**
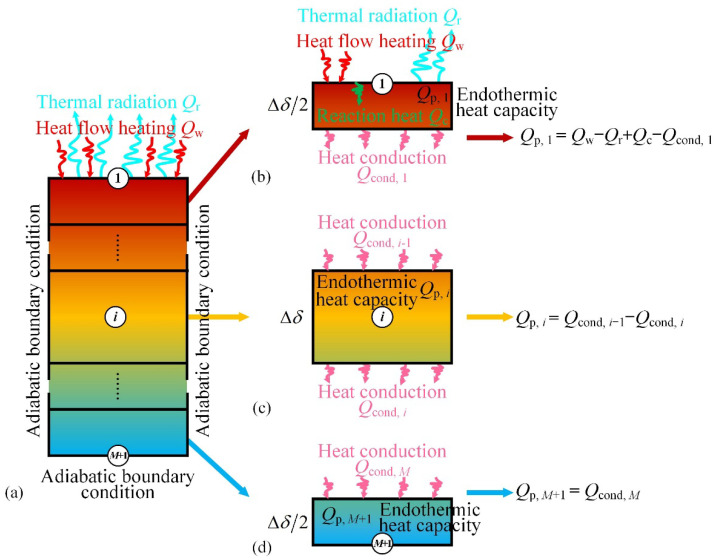
Schematic diagram of heat transfer: (**a**) boundary condition; (**b**) element 1; (**c**) element *i*; (**d**) element *M* + 1.

Equation (20) describes the change of internal energy due to heat conduction. The sample is spatially divided into *M* + 1 elements, as shown in Figure 4a. Element 1 is the upper surface element of the sample, which includes the endothermic heat capacity *Q*_p_, energy of heat-flow heating *Q*_w_, thermal radiation *Q*_r_, chemical reaction heat *Q*_c_, and heat conduction *Q*_cond_. As shown in Figure 4b, the energy conservation equation for element 1 is given by [50]:

*Q*_p,1_ = *Q*_w_ − *Q*_r_ + *Q*_c_ − *Q*_cond,1_, (21)

where

(22) $$Q_{p,1}=C_{p}(T_{1}(t)-T_{1}(t-\Delta t))\rho S\frac{\Delta \delta }{2},$$

*Q*_w_ = *q*_w_*S*Δ*t*, (23)

(24) $$q_{w}=\psi q_{\mathrm{cold}}(1-\frac{h_{w}}{h_{\mathrm{re}}}),$$

(25) $$\psi =1-0.62(\frac{29}{M_{\mathrm{CO}}}{)}^{0.26}{\dot{m}}_{\mathrm{CO}}\frac{h_{\mathrm{re}}}{q_{\mathrm{cold}}},$$

(26) $$Q_{r}=\varepsilon \sigma T_{w}^{4}S\Delta t,$$

*Q*_c_ = Δ*H*_C_*J*_C_*M*_C_*S*Δ*t*, (27)

(28) $$Q_{\mathrm{cond},1}=\frac{T_{1}\left(t\right)-T_{2}\left(t\right)}{\Delta \delta }\lambda S\Delta t,$$

where *q*_w_, *q*_cold_, *h*_w_, and *h*_re_ are the actual heat flux considering the thermal blockade effect, the cold wall heat flux, the wall enthalpy, and the recovery enthalpy of the boundary layer, respectively; *ψ*, *M*_CO_, ${\dot{m}}_{\mathrm{CO}}$, *M*_C_, Δ*H*_C_, *ε*, *σ*, *T*_w_ and *S* are the mass ejector factor, molar mass of CO, mass flux of CO, molar mass of C, reaction heat, radiation coefficient, Stefan–Boltzmann constant, wall temperature, and the area of the sample section, respectively. Thus, the energy conservation equation for element 1 can also be written as:

(29) $$C_{p}(T_{1}(t)-T_{1}(t-\Delta t))\rho S\frac{\Delta \delta }{2}=\psi q_{\mathrm{cold}}(1-\frac{h_{w}}{h_{\mathrm{re}}})S\Delta t-\varepsilon \sigma T_{w}^{4}S\Delta t+\Delta H_{C}J_{C}M_{C}S\Delta t\;\;-\frac{T_{1}\left(t\right)-T_{2}\left(t\right)}{\Delta \delta }\lambda S\Delta t,$$

There are only endothermic heat capacity and the heat conduction in the internal elements of the material. As shown in Figure 4c, the energy conservation equation for elements 2 to *M* is given by

*Q*_p,*i*_ = *Q*_cond,*i*−1_ − *Q*_cond,*i*_, (30)

(31) $$C_{p}(T_{i}(t)-T_{i}(t-\Delta t))\rho S\Delta \delta =\frac{T_{i-1}\left(t\right)-T_{i}\left(t\right)}{\Delta \delta }\lambda S\Delta t-\frac{T_{i}\left(t\right)-T_{i+1}\left(t\right)}{\Delta \delta }\lambda S\Delta t.$$

Because of the adiabatic boundary condition, the heat transfer between element *M* + 1 and the outside environment is ignored. As shown in Figure 4d, the energy conservation equation for element *M* + 1 can be expressed as:

*Q*_p,*M*+1_ = *Q*_cond,*M*_, (32)

(33) $$C_{p}(T_{M+1}(t)-T_{M+1}(t-\Delta t))\rho S\frac{\Delta \delta }{2}=\frac{T_{M}\left(t\right)-T_{M+1}\left(t\right)}{\Delta \delta }\lambda S\Delta t.$$

### 2.4. Moving Boundary

As the surface temperature of the carbon/carbon composite increases continuously during the ablation process, the oxidation of carbon on the surface leads to a reduction of the ablation material thickness. The reduction in material thickness causes the change in the heat transfer. Consequently, it is necessary to consider the boundary movement caused by ablation in the one-dimensional thermal response model. The thickness of the composite and elements at time t can be written as:

*δ*(*t*) = *δ*(*t* − Δ*t*) − *v*_micro_(*t*)Δ*t*, (34)

(35) $$\Delta \delta (t)=\frac{\delta \left(t-\Delta t\right)-v_{\mathrm{micro}}\left(t\right)\Delta t}{M}.$$

The temperature of each element considering the boundary movement is corrected by the interpolation method.

## 3. Calculation Process

**Figure 5:**
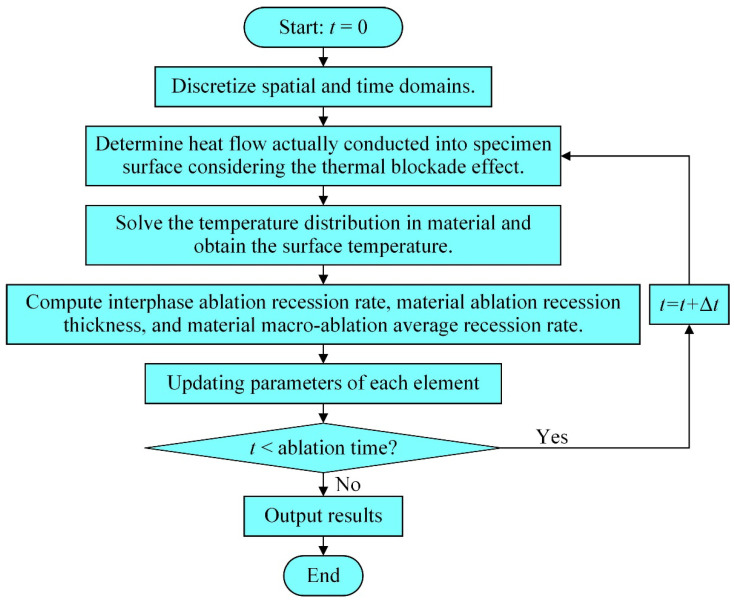
Solution flow chart for ablation performance of carbon/carbon composite.

The heat transfer and surface recession should be considered when solving for the temperature distribution in the carbon/carbon composite. Figure 5 shows the flow chart for the solution of the carbon/carbon composite ablation model using MATLAB. The first step is to discretize the spatial and time domains, as described in Section 2.3. Then, based on the initial boundary conditions, the actual heat flux on the material surface *q*_w_ is calculated. In the third step, the temperature distribution at time *t* is solved using the energy conservation equations in the one-dimensional thermal response model. Because the energy equations are written as a block tridiagonal matrix, the chasing method is used to solve this problem. Subsequently, the surface temperature can be obtained. The fourth step is to compute the micro-ablation recession rate *v*_micro_, the ablation recession thickness of the material *δ*_ac_, and the macro-ablation recession rate of the material *v*_macro_. The parameters of each element are then updated in the fifth step. If the cumulative time has not reached the set requirements, steps 2–5 are repeated and *t* is set to *t* + Δ*t*; otherwise, the results are output.

Table 1 displays the relevant parameters of the carbon/carbon composite. We additionally assume that the boundary layer thickness at the stagnation point *δ*_c_ is 0 m [40]. Using the above solution procedure, the material surface and back surface temperatures, the interphase micro-ablation recession rate, and the macro-ablation average recession of carbon/carbon composites (Φ 24 mm × 100 mm) ablated for 30 s were calculated. The heat flux *q*_cold_, gas recovery enthalpy at the boundary layer *h*_re_, and stagnation point pressure *P*_s_ were respectively set to 18,338 kW·m^−2^, 8051 kJ·kg^−1^, and 0.598 MPa for the calculation. The results and discussion are shown in Section 4.

## 4. Results and Discussion

### 4.1. Model Validation

**Figure 6:**
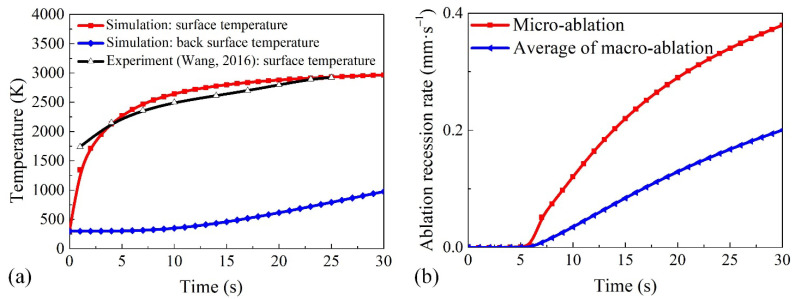
(**a**) Ablation temperature (Reprinted/adapted with permission from Ref. [25]. 2016, Elsevier.) and (**b**) ablation recession rate plotted against the ablation time.

Table 2 and Figure 6 show a comparison between the calculated and experimental results for the ablation performance under an oxyacetylene flame. The macro-ablation recession rate and surface temperature predicted by the current model are consistent with the experimental results in Reference [25]. As shown in Figure 6a, the surface temperature of the material increased rapidly to approximately 2000 K under the action of the heat flux. The surface temperature and back surface temperature of the material after ablation for 30 s were 2967 K and 976 K, respectively. As shown in Figure 6b, the micro- and macro-ablation recession rate of the material increased slowly during the first 6 s of ablation because of the low surface temperature and transient-state ablation. After ablation for 6 s, the micro- and macro-ablation recession rates increased significantly along with the surface temperature. This is because the surface temperature is still low during the first 6 s of ablation, and the ablation morphology of the fiber has not yet reached its steady state during the first 6 s of ablation and shows a plateau. The micro- and macro-ablation behaviors are thus described by the recession rate of the fiber, which is relatively small. The final macro-ablation recession rate of the material reached after ablation for 30 s was 0.2003 mm/s. The prediction ablation recession rate of this work is greater than that of the traditional model, which is approximately 6.5%. The reason is that the ablation velocity direction of the fiber is not parallel to the macroscopic recession direction, and this factor is considered in our model. The rationality of this result can be explained in Figure 3. Therefore, it is necessary to consider the characteristics of fiber morphology to modify the thermochemical ablation model.

### 4.2. Factors Affecting Micro-Ablation Recession Rate

**Figure 7:**
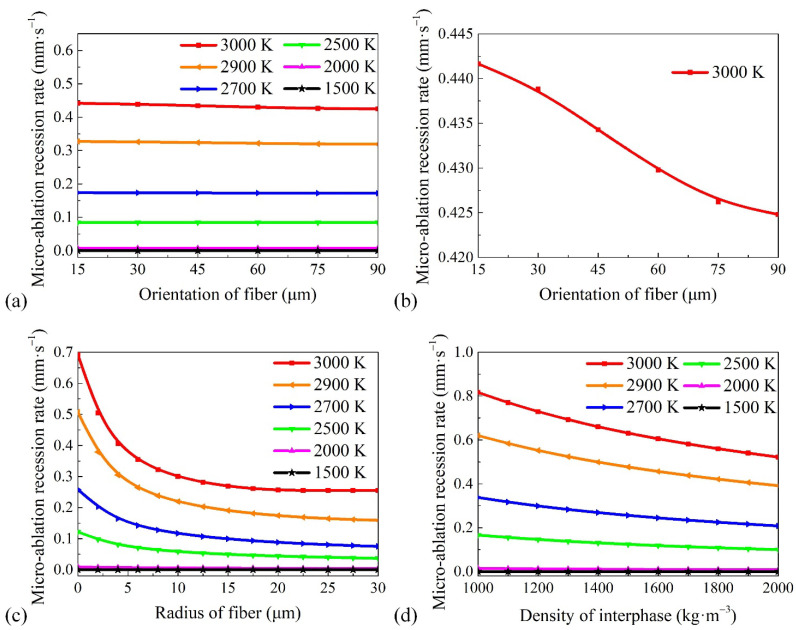
Variation of micro-ablation recession rate with (**a**) fiber orientation; (**b**) fiber orientation when the temperature is 3000 K; (**c**) fiber radius; and (**d**) interphase density at different temperatures (*T* = 1500, 2000, 2500, 2700, 2900, 3000 K).

The surface morphology reaches a steady state on the microscopic scale when the ablation surface temperature is given. When the carbon fibers are not platform-shaped, it is difficult to accurately find a recession velocity parallel to the object surface on the fiber surface. Thus, the micro-ablation recession rate of the carbon fibers cannot be directly used to describe macro-ablation recession. However, due to the interphase in the model assumed to be parallel to the object plane, the micro-ablation recession rate of the interphase is a crucial parameter that directly reflects the magnitude of the macro-ablation average recession rate. Thus, the influence of the fiber orientation, fiber radius, interphase density, and ablation surface temperature on the micro-ablation recession rate of the interphase was studied, and the results are shown in Figure 7. The results indicate that the interphase micro-ablation recession rate changes slowly with an increase in the fiber orientation, radius, and interphase density when the temperature is lower than 2000 K. The material reactivity at low temperatures is lower than that at high temperatures, and the change in the micro-ablation recession rate induced by the change in the influencing factors is not significant.

As shown in Figure 7a, the fiber orientation does not have a significant influence on the micro-ablation recession rate at different temperatures. The micro-ablation recession rate was reduced by only approximately 4% over the fiber orientation range studied, as shown in Figure 7b. This is because the fiber height difference induced by the fiber orientation is not significant, as has been reported in Reference [40]. However, 4% optimization is also very important and cannot be ignored in the aerospace field.

As shown in Figure 7c, the micro-ablation recession rate is very sensitive to changes in the fiber radius, especially when the fiber radius is small and the temperature is high. The fiber height in the steady state increases with the fiber radius. This causes the oxygen concentration on the interphase surface to decrease. Thus, the micro-ablation recession rate decreases significantly as the fiber radius increases. At 3000 K, the micro-ablation recession rate of the ablation material without carbon fibers is much greater than of the material with carbon fibers. The results also indicate that the interphase micro-ablation recession rate does not change with an increase in the fiber radius, when the temperature is 3000 K and the fiber radius is larger than 22.5 μm. The reason is that the steady-state ablation morphology of the fiber presents a platform shape, and the fiber height no longer increases with an increase in the fiber radius in this case.

An increase in the interphase density indicates a greater number of carbon atoms per unit volume. If the reactivity on the interphase surface is constant, it takes longer for the dense interphase to decay to the same thickness. Thus, the micro-ablation recession rate decreases with an increase in the interphase density, as shown in Figure 7d. However, the decrease in the micro-ablation recession rate of the interphase causes the steady-state morphology of the fiber to decrease. The oxygen concentration on the surface of the interphase increases, resulting in an increase in the micro-ablation recession rate. This reduces the decrease in the micro-ablation rate.

In summary, the anti-ablation efficiency of the material can be enhanced by increasing the fiber radius and the interphase density.

### 4.3. Factors Affecting Macro-Ablation Recession Rate

**Figure 8:**
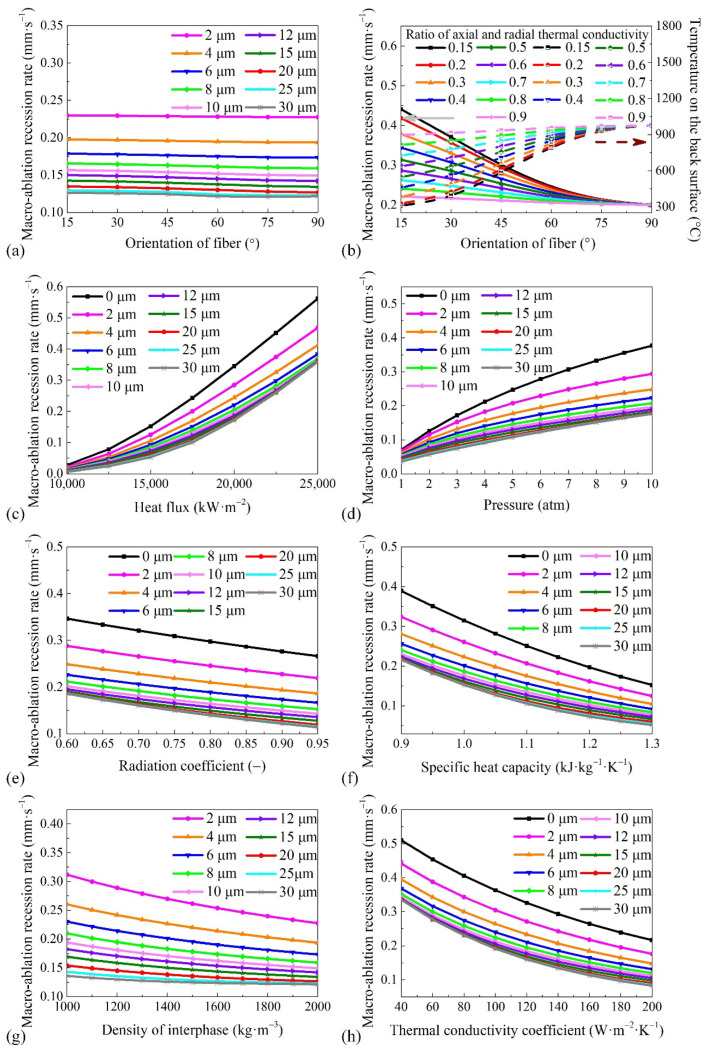
Variation of macro-ablation recession rate with (**a**) orientation of fiber at different fiber radii (*R* = 0, 2, 4, 6, 8, 10, 15, 20, 30 μm); (**b**) orientation of fiber at different radial and axial thermal conductivity ratios (*λ*_1_/*λ*_2_ = 0.15, 0.2, 0.3, 0.4, 0.5, 0.6, 0.7, 0.8, 0.9); (**c**) heat flux; (**d**) pressure; (**e**) radiation coefficient; (**f**) specific heat capacity; (**g**) density of interphase; and (**h**) thermal conductivity coefficient.

The macro-ablation average recession rate is the most important indicator used in engineering to evaluate the properties of ablation materials, which are affected by the heat flux, pressure, radiation coefficient, specific heat capacity, fiber radius, fiber orientation, interphase density, and thermal conductivity of the material. Thus, the factors influencing the macro-ablation average recession rate are investigated in this section, and the results are shown in Figure 8.

The relationship between the macro-ablation average recession rate and the fiber orientation is shown in Figure 8a, in which the change in the thermal conductivity coefficient is not considered. The fiber orientation has little effect on the steady-state morphology of the fiber. Thus, the macro-ablation average recession rate is not significantly changed by the variation of the fiber orientation, similar to the micro-ablation recession rate. However, this result is inconsistent with the previous experimental results. Figure 8b shows the results of considering the change of thermal conductivity coefficients with fiber orientation. The results indicate that the fiber orientation has a significant influence on the macro-ablation average recession rate when the ratio of the radial and axial thermal conductivity coefficients *λ*_1_/*λ*_2_ is considered. The results explain the reason why the fiber orientation affects the ablation performance. The key reason for this is because the fiber orientation affects the thermal conductivity of the material along the thickness direction, which in turn modifies the material surface temperature and the reactivity. The lowest macro-ablation average recession rate occurs in the direction of the maximum thermal conductivity coefficient. However, increasing the fiber orientation angle increases the temperature of the back surface. Hence, a proper arrangement of fibers can effectively improve the ablation resistance of the material, albeit at the cost of an increased back surface temperature.

The material and environment parameters in Figure 8c are the same as those in Section 3, except for the heat flux and fiber radius. The results indicate that the average macro-ablation recession rate increases rapidly with the heat flux. The reason is that a larger heat flux heats the surface of the material to a higher temperature faster and results in higher reactivity.

As shown in Figure 8d, the effect of pressure on the macro-ablation average recession rate is similar to that of the heat flux. The pressure affects the ablation rate by changing the oxygen concentration and the diffusion rate. A higher pressure increases the oxygen concentration in the environment but reduces the diffusion coefficient.

As shown in Figure 8e–g, the macro-ablation average recession rate decreases steadily with the increase of the radiation coefficient, specific heat capacity, interphase density, and thermal conductivity coefficient. Hence, the ablation performance of the material can be improved by increasing its radiation coefficient, specific heat capacity, interphase density, or thermal conductivity coefficient. The parameters that influence the macro-ablation average recession rate within the context of parameter changes discussed are, in deceasing order of significance, the specific heat capacity, thermal conductivity coefficient, radiation coefficient, and interphase density. The mechanism through which an increase in the thermal conductivity coefficient, specific heat capacity, or radiation coefficient reduces the ablation rate is mainly the reduction of the surface temperature. Therefore, the reactivity of each component is reduced. The effect of the interphase density on the macro-ablation average recession rate is similar to that of the micro-ablation recession rate, which has been discussed in Section 4.1.

Meanwhile, the influence of the fiber radius on the macro-ablation average recession rate is significant, as shown in Figure 8c–g. For example, the macro-ablation average recession rate of the ablation material without carbon fibers is approximately 1.57 times greater than that of the same material with 30 μm radius carbon fibers under high heat flux (25,000 kW·m^−2^), and it is approximately 4.71 times greater than that of the same material with 30 μm radius carbon fibers under low heat flux (10,000 kW·m^−2^). In addition, it is worth pointing out that when the fiber radius is larger than 20 μm, the impact of the fiber radius on the macro-ablation average recession rate is not significant. This is because although increasing the fiber radius still increases the fiber height and decreases the oxygen concentration on the interphase surface when the fiber radius is greater than 20 μm, the influence of the increased radius is greatly diminished. This implies that it is difficult to efficiently increase the ablation performance of a material by increasing the fiber radius when the fiber radius is greater than 20 μm.

**Figure 9:**
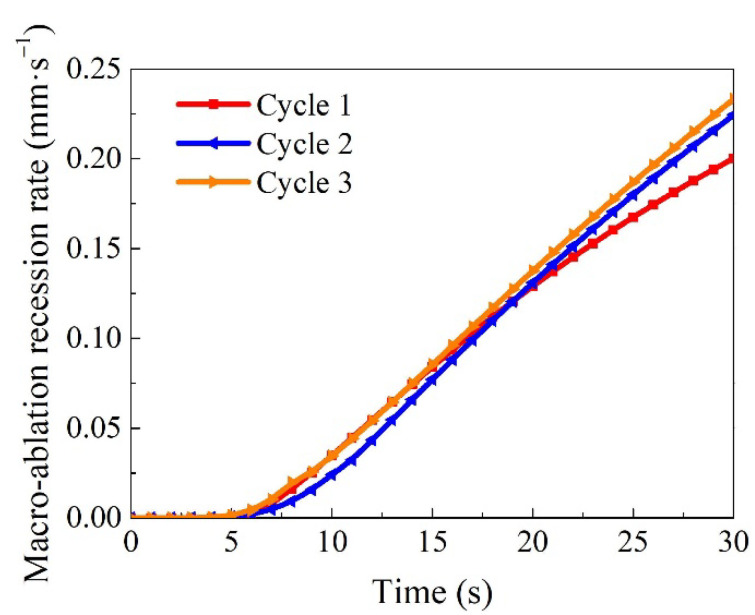
Variation of macro-ablation recession rate with ablation time at different cycle times.

In addition, the relationship between the macro-ablation recession rate of cyclic ablation and time is shown in Figure 9. The results indicate that the macro-ablation recession rate of ablation for 30 s increases rapidly with the increase of the number of cycles. The macro-ablation recession rates of cycle 1, cycle 2, and cycle 3 are 0.200, 0.224, and 0.233 mm/s respectively. The reason is that the surface of the sample after ablation is rougher than that of the original material, which increases the oxidation rate. Meanwhile, the thickness of the sample after ablation decreases, resulting in a higher surface temperature and an increased oxidation rate.

## 5. Conclusions

A thermochemical ablation model of microstructure-based carbon/carbon composites was established to investigate the effects of the fiber radius, fiber orientation, interphase density, surface temperature, radiation coefficient, specific heat capacity, and thermal conductivity coefficient on the ablation recession rate. The predicted results from this model are consistent with the experimental results. The differences in the thermal conductivity coefficient caused by the fiber orientation can significantly affect the ablation performance of the material. Thus, a suitable arrangement of fibers can easily improve the anti-ablation ability of the material. The ablation recession rate of the ablation material without carbon fibers is much greater than that of the material containing carbon fibers. The anti-ablation efficiency of the material can be improved by increasing the radiation coefficient, specific heat capacity, interphase density, and thermal conductivity coefficient of the material. The parameters that influence the macro-ablation average recession rate are, in deceasing order of significance, the specific heat capacity, thermal conductivity coefficient, radiation coefficient, and interphase density. The influence of the fiber radius on the ablation recession rate is not significant when the fiber radius is sufficiently large (above 20 μm). The ablation resistance decreases with increases in the number of ablation cycles. The thermochemical ablation model in this work offers a guide for the design and preparation of better ablation materials.

## Figures and Tables

**Table 1:** Properties of carbon/carbon composite.

Parameters	Values
Pre-exponential factor of fiber, *A*_f_ (m·s^−1^) [51]	2002.2 × *T*
Pre-exponential factor of interphase, *A*_i_ (m·s^−1^)	241,000
Activation energy of fiber, *Ea*_f_ (J·mol^−1^) [51]	353,365
Activation energy of interphase, *Ea*_i_ (J·mol^−1^) [44]	245,000
Density of fiber and interphase, *ρ*_f_ and *ρ*_i_ (kg·m^−3^) [14]	2000
Thermal conductivity coefficient, *λ* (W·m^−1^·K^−1^) [52]	151.55
Specific heat capacity, *C*_p_ (kJ·kg^−1^·K^−1^) [25]	0.997 + 2.93 × 10^−5^ × *T*
Radiation coefficient, ε (-) [53]	0.9

**Table 2:** Comparison between the predicted and experimentally measured jet arc ablation performance.

*q*_cold_/ (kW·m^−2^)	*h*_re_/ (kJ·kg^−1^)	*p*_s_/ (MPa)	Surface Temperature at 25 s *T*_w_/(K)			Ablation Recession Rate at 30 s *v*_−∞_/ (mm·s^−1^)		
			Predicted Results of This Work	Predicted Results of Reference	Experimental Results of Reference	Predicted Results of This Work	Predicted Results of Reference	Experimental Results of Reference
18,338 [25]	8051 [25]	0.598 [25]	2922	3046 [25]	2923 [25]	0.2003	0.188 [25]	0.201 [25]

## Data Availability

Data is contained within the article.

## References

[1] Cao J., Chen J.B., Wang X.B., Wen J.B. (2021). Tribology and anti-ablation properties of SiC-VN-MoS2/Ta composite coatings on carbon/carbon composites from 25 to 800 °C. Materials.

[2] Lv B.W., Mucke R., Fan X.L., Wang T.J., Guilon O., Vaben R. (2018). Sintering resistance of advanced plasma-sprayed thermal barrier coatings with strain-tolerant microstructures. J. Eur. Ceram. Soc..

[3] Lv B.W., Fan X.L., Li D.J., Wang T.J. (2018). Towards enhanced sintering resistance: Air-plasma-sprayed thermal barrier coating system with porosity gradient. J. Eur. Ceram. Soc..

[4] Wang T.J. (1992). Unified CDM model and local criterion for ductile fracture-I. Unified CDM model for ductile fracture. Eng. Fract. Mech..

[5] Ma L.S., Wang T.J. (2004). Relationships between axisymmetric bending and buckling solutions of FGM circular plates based on third-order plate theory and classical plate theory. Int. J. Solids Struct..

[6] Ma L.S., Wang T.J. (2003). Nonlinear bending and post-buckling of a functionally graded circular plate under mechanical and thermal loadings. Int. J. Solids Struct..

[7] Shao Z.S., Wang T.J. (2006). Three-dimensional solutions for the stress fields in functionally graded cylindrical panel with finite length and subjected to thermal/mechanical loads. Int. J. Solids Struct..

[8] Lv B.W., Mücke R., Zhou D.P., Fan X.L., Wang T.J., Guillon O., Vaßen R. (2019). A constitutive model for the sintering of suspension plasma-sprayed thermal barrier coating with vertical cracks. J. Am. Ceram. Soc..

[9] Li B., Fan X.L., Zhou K., Wang T.J. (2018). A semi-analytical model for predicting stress evolution in multilayer coating systems during thermal cycling. Int. J. Mech. Sci..

[10] Fang H., Li J., Chen H., Liu B., Huang W., Liu Y., Wang T.J. (2016). Radiation induced degradation of silica reinforced silicone foam: Experiments and modeling. Mech. Mater..

[11] Song Y., Lv Z., Liu Y., Zhuan X., Wang T.J. (2015). Effect of coating spray speed and convective heat transfer on transient thermal stress in thermal barrier coating system during the cooling process of fabrication. Appl. Surf. Sci..

[12] Kang B.R., Kim H.S., Oh P.Y., Lee J.M., Lee H.I., Hong S.M. (2019). Characteristics of ZrC barrier coating on SiC-coated carbon/carbon composite developed by thermal spray process. Materials.

[13] Ye C., Huang D., Li B.L., Yang P.J., Liu J.S., Wu H., Yang J.X., Li X.K. (2019). Ablation behavior of the SiC-coated three-dimensional highly thermal conductive mesophase-pitch-based carbon-fiber-reinforced carbon matrix composite under plasma flame. Materials.

[14] Jin X.C., Fan X.L., Lu C.S., Wang T.J. (2018). Advances in oxidation and ablation resistance of high and ultra-high temperature ceramics modified or coated carbon/carbon composites. J. Eur. Ceram. Soc..

[15] Levet C., Lachaud J., Ducamp V., Memes R., Couzi J., Mathiaud J., Gillard A.P., Weisbecker P., Vignoles G.L. (2021). High-flux sublimation of a 3D carbon/carbon composite: Surface roughness patterns. Carbon.

[16] Fan X.L., Jiang P., Li B., Jin X.C., Zhao Y. (2017). Experimental and numerical evaluation of the ablation process of carbon/carbon composites using high velocity oxygen fuel system. Adv. Mater. Sci. Eng..

[17] Mckee D.W. (1987). Oxidation behavior and protection of carbon/carbon composites. Carbon.

[18] Mcmanus H.L.N., Springer G.S. (1992). High temperature thermomechanical behavior of carbon-phenolic and carbon-carbon composites, I. analysis. J. Compos. Mater..

[19] Guo Y.J., Dai G.Y., Gui Y.W., Tong F.L., Qiu B., Liu B. (2014). A dual platform theory for carbon-based material oxidation with reaction-diffusion rate controlled kinetics. ACTA Aerodyn. Sin..

[20] Yin T.T., Zhang Z.W., Li X.F., Feng X., Feng Z.H., Wang Y., He L.H., Gong X.L. (2014). Modeling ablative behavior and thermal response of carbon/carbon composites. Comput. Mater. Sci..

[21] Li H.Z., Li S.G., Wang Y.C. (2011). Prediction of effective thermal conductivities of woven fabric composites using unit cells at multiple length scales. J. Mater. Res..

[22] Liu Z.G., Zhang H.G., Lu Z.X., Li D.S. (2007). Investigation on the thermal conductivity of 3-dimensional and 4-directional braided composites. Chin. J. Aeronaut..

[23] Mei Z.S., Shi C.Y., Fan X.L., Wang X.B. (2020). Numerical simulation of hypersonic reentry flow field with gas-phase and surface chemistry models. Mater. Today Commun..

[24] Mei Z.S., Shi C.Y., Fan X.L., Wang X.B. (2020). Coupled simulation for reentry ablative behavior of hypersonic vehicles. IOP Conf. Ser. Mater. Sci. Eng..

[25] Wang C. (2016). Numerical analyses of ablative behavior of C/C composite materials. Int. J. Heat Mass Transf..

[26] Vignoles G.L., Lachaud J., Aspa Y., Goyheneche J.M. (2009). Ablation of carbon-based materials: Multiscale roughness modelling. Compos. Sci. Technol..

[27] Chen Z.F., Fang D., Miao Y.L., Yan B. (2008). Comparison of morphology and microstructure of ablation centre of C/SiC composites by oxy-acetylene torch at 2900 and 3550 °C. Corros. Sci..

[28] Zeng Y., Xiong X., Li G.D., Chen Z.K., Sun W., Wang D.N., Wang Y.L. (2013). Effect of fiber architecture and density on the ablation behavior of carbon/carbon composites modified by Zr-Ti-C. Carbon.

[29] Farhan S., Li K.Z., Guo L.J., Guo Q.M., Lan F.T. (2010). Effect of density and fibre orientation on the ablation behaviour of carbon-carbon composites. New Carbon Mater..

[30] Gao G.X., Zhang Z.C., Zheng Y.S., Jin Z.H. (2010). Effect of fiber orientation angle on thermal degradation and ablative properties of short-fiber reinforced EPDM/NBR rubber composites. Polym. Compos..

[31] Shi Y.A., He L.X., Qiu B., Zeng L., Geng X.R., Wei D. (2016). Multiscale heat transfer analysis of Z-directional carbon fiber reinforced braided composites. Acta Aeronaut. Astronaut. Sin..

[32] Zhang B., Li X.D. (2018). Thermal response of a 4D carbon/carbon composite with volume ablation: A numerical simulation study. Appl. Compos. Mater..

[33] Liu Z.G., Zhang J.S., Han J.C. (2005). Results analyzed of surper high temperature thermal chemical ablation for carbon-based composite material. Carbon.

[34] Liu Z.G., Han J.C., Du S.Y., Zhang W. (2006). High temperature thermal chemical ablative calculation for carbon composite materials by minimization of energy function. Acta Mater. Compos. Sin..

[35] Guo W.M., Xiao H.N., Yasuda E., Cheng Y. (2006). Oxidation kinetics and mechanisms of a 2D-C/C composite. Carbon.

[36] Qin F., Peng L.N., He G.Q., Li J. (2013). Oxidation kinetics and mechanisms of four-direction carbon/carbon composites and their components in carbon dioxide at high temperature. Corros. Sci..

[37] Meng S.H., Zhou Y.J., Xie W.H., Yi F.J., Du S.Y. (2016). Multiphysics coupled fluid/thermal/ablation simulation of carbon/carbon composites. J. Spacecr. Rocket..

[38] Han J.C., He X.D., Du S.Y. (1995). Oxidation and ablation of 3D carbon-carbon composite at up to 3000 °C. Carbon.

[39] Lachaud J., Aspa Y., Vignoles G.L., Goyheneche J.M. 3D modeling of thermochemical ablation in carbon-based materials: Effect of anisotropy on surface roughness onset. Proceedings of the 10th International Symposium on Materials in a Space Environment.

[40] Lachaud J., Aspa Y., Vignoles G.L. (2008). Analytical modeling of the steady state ablation of a 3D C/C composite. Int. J. Heat Mass Transf..

[41] Zhang B., Li X.D. (2018). Numerical simulation of thermal response and ablation behavior of a hybrid carbon/carbon composite. Appl. Compos. Mater..

[42] Liu N., Yang Q.S. Micromechanical modeling and numerical simulation of ablation of 3D C/C composites. Proceedings of the 13th International Conference on Fracture.

[43] Li W., Fang G.D., Li W.J., Liang J., Li M. (2019). Role of mesoscopic features on thermochemical ablative behavior of 3D C/C braided composites. Int. J. Heat Mass Transf..

[44] Bacos M.P., Cochon J.L., Dorvaux J.M., Lavigne O. (2000). C/C composite oxidation model: II. Oxidation experimental investigations. Carbon.

[45] Lachaud J., Bertrand N., Vignoles G.L., Bourget G., Rebillat F., Weisbecker P. (2007). A theoretical/experimental approach to the intrinsic oxidation reactivities of C/C composites and of their components. Carbon.

[46] Chapman S., Cowling T.G. (1970). The Mathematical Theory of Non-Uniform Gases.

[47] Inger G.R. (2000). Scaling nonequilibrium-reacting flows: The legacy of Gerhard Damköhler. J. Spacecr. Rockets.

[48] Shi S.B., Li L.J., Liang J., Tang S. (2016). Surface and volumetric ablation behaviors of SiFRP composites at high heating rates for thermal protection applications. Int. J. Heat Mass Transf..

[49] Chen Y.K., Milos F.S. (2001). Two-dimensional implicit thermal response and ablation program for charring materials. J. Spacecr. Rocket..

[50] Shi S.B., Liang J., Yi F.J., Fang G.D. (2013). Modeling of one-dimensional thermal response of silica-phenolic composites with volume ablation. J. Compos. Mater..

[51] Swann R.T., Pittman C.M., Smith J.C. (1965). One-Dimensional Numerical Analysis of the Transient Response of Thermal Protection Systems.

[52] Jie C., Xiang X., Peng X. (2009). Thermal conductivity of unidirectional carbon/carbon composites with different carbon matrixes. Mater. Des..

[53] Dimitrienko Y.I. (1999). Thermomechanics of Composites under High Temperatures.

